# Reslice3Dto2D: Introduction of a software tool to reformat 3D volumes into reference 2D slices in cardiovascular magnetic resonance imaging

**DOI:** 10.1186/s13104-024-06931-4

**Published:** 2024-09-17

**Authors:** Darian Viezzer, Maximilian Fenski, Thomas Hiroshi Grandy, Johanna Kuhnt, Thomas Hadler, Steffen Lange, Jeanette Schulz-Menger

**Affiliations:** 1 Charité – Universitätsmedizin Berlin, Corporate Member of Freie Universität Berlin and Humboldt-Universität zu Berlin, ECRC Experimental and Clinical Research Center, Lindenberger Weg 80, 13125 Berlin, Germany; 2 Working Group on Cardiovascular Magnetic Resonance, Experimental and Clinical Research Center, a joint cooperation between the Charité – Universitätsmedizin Berlin and the Max-Delbrück-Center for Molecular Medicine, Berlin, Germany; 3 DZHK (German Centre for Cardiovascular Research), Partner Site Berlin, Berlin, Germany; 4 Department of Cardiology and Nephrology, Helios Hospital Berlin-Buch, Berlin, Germany; 5 Faculty for Computer Sciences, Hochschule Darmstadt (University of Applied Sciences), Darmstadt, Germany

**Keywords:** 3D, 2D, Cardiovascular Magnetic Resonance, CMR, Reference slice position, Post-processing, Quantification

## Abstract

**Objective:**

Cardiovascular magnetic resonance enables the quantification of functional and morphological parameters with an impact on therapeutical decision making. While quantitative assessment is established in 2D, novel 3D techniques lack a standardized approach. Multi-planar-reformatting functionality in available software relies on visual matching location and often lacks necessary functionalities for further post-processing. Therefore, the easy-to-use Reslice3Dto2D software tool was developed as part of another research project to fill this gap and is now introduced with this work.

**Results:**

The Reslice3Dto2D reformats 3D data at the exact location of a reference slice with a two-step-based interpolation in order to reflect in-plane discretization and through-plane slice thickness including a slice profile selection. The tool was successfully validated on an artificial dataset and tested on 119 subjects with different underlying pathologies. The exported reformatted data could be imported into three different post-processing software tools. The quantified image sharpness by the Frequency Domain Image Blur Measure was significantly decreased by around 40% on rectangular slice profiles with 7 mm slice thickness compared to 0 mm due to partial volume effects. Consequently, Reslice3Dto2D enables the quantification of 3D data with conventional post-processing tools as well as the comparison of 3D acquisitions with their established 2D version.

**Supplementary Information:**

The online version contains supplementary material available at 10.1186/s13104-024-06931-4.

## Introduction

In current guidelines cardiovascular magnetic resonance (CMR) is a recommended imaging modality for the characterization of cardiovascular diseases [1–3]. The quantification of functional and morphological parameters is highly relevant in clinical decision making [1, 2].

Due to technical restrictions, mainly two dimensional (2D) acquisition methods were available in the past [4] while novel technical developments enable three dimensional (3D) acquisitions for several sequence types like CINE [4], angiography [5], flow [6], Late Gadolinium Enhancement (LGE) [5, 7, 8], perfusion imaging [9], parametric mapping [10] and fat/water imaging [8]. The advantages of 3D acquisitions compared to 2D include the omission of a complex slice positioning during examination [4], the possibility to cover of the whole heart [4], an improved diagnosis of cardiovascular diseases with complex anatomic arrangement [3], a decreased impact of partial volume effects [7] and the potential of simultaneous sequence acquisition [8, 10].

Nonetheless, conventional and established 2D sequences provide the gold standard in most clinical settings [7] and represent the quantitative and qualitative validation for novel 3D sequences [4, 7]. As the post-processing of 3D data is neither standardized nor routinely available, a reformatting is currently necessary to enable usage of conventional 2D post-processing tools. Analysis software products offer multi-planar-reformatting (MPR) functionalities that rely on manual or basic oriented (axial, sagittal or coronal) 2D plane definitions [4, 11]. Some of these tools lack DICOM [12] export or processing functionalities and thus limits the post-processing capabilities. Furthermore, a difference in the actual scanner produced slice profile and an ideal rectangular shaped slice profile has been reported [13]. The inclusion of slice profile and respective slice thickness enables to reproduce more accurate scanner behavior. While open-source solutions, like 3D slicer [11], offer desired functionalities either intrinsic, by extension modules or by the possibility of self-developing extensions, the necessary expertise for the usage represents an obstacle.

Hence, this work aims to introduce our self-developed and easy-to-use Reslice3Dto2D software as an intermediate processing tool for the clinical research of novel 3D acquisition methods. The tool was developed with a focused use-case on CMR and enables the reformatting of 3D data to the exact location of reference 2D acquisitions including slice thickness adjustments, slice profile options and a DICOM [12] export functionality.

## Methods

As this work introduces software, the implementation of the Reslice3Dto2D tool is described first, followed by a description of its validation and testing.

### Implementation

The Reslice3Dto2D tool was fully implemented in Python (Version 3.8, Python Software Foundation) and includes a graphical user interface (GUI). The source-code is provided as supplemental material S1 and made publicly available [14]. Installation details are provided in the README.md of the source-code and user guidance is described in detail in the user manual of the supplemental material S2. The user manual lists also DICOM [12] tags that are a must for the functionality of the Reslice3Dto2D tool. Future maintenance of the source-code is not guaranteed, but re-usage and further development is explicitly allowed. For Windows and macOS, packed executable Reslice3Dto2D files were publicly provided for direct usage without the necessity to install anything [14].

**Fig. 1:**
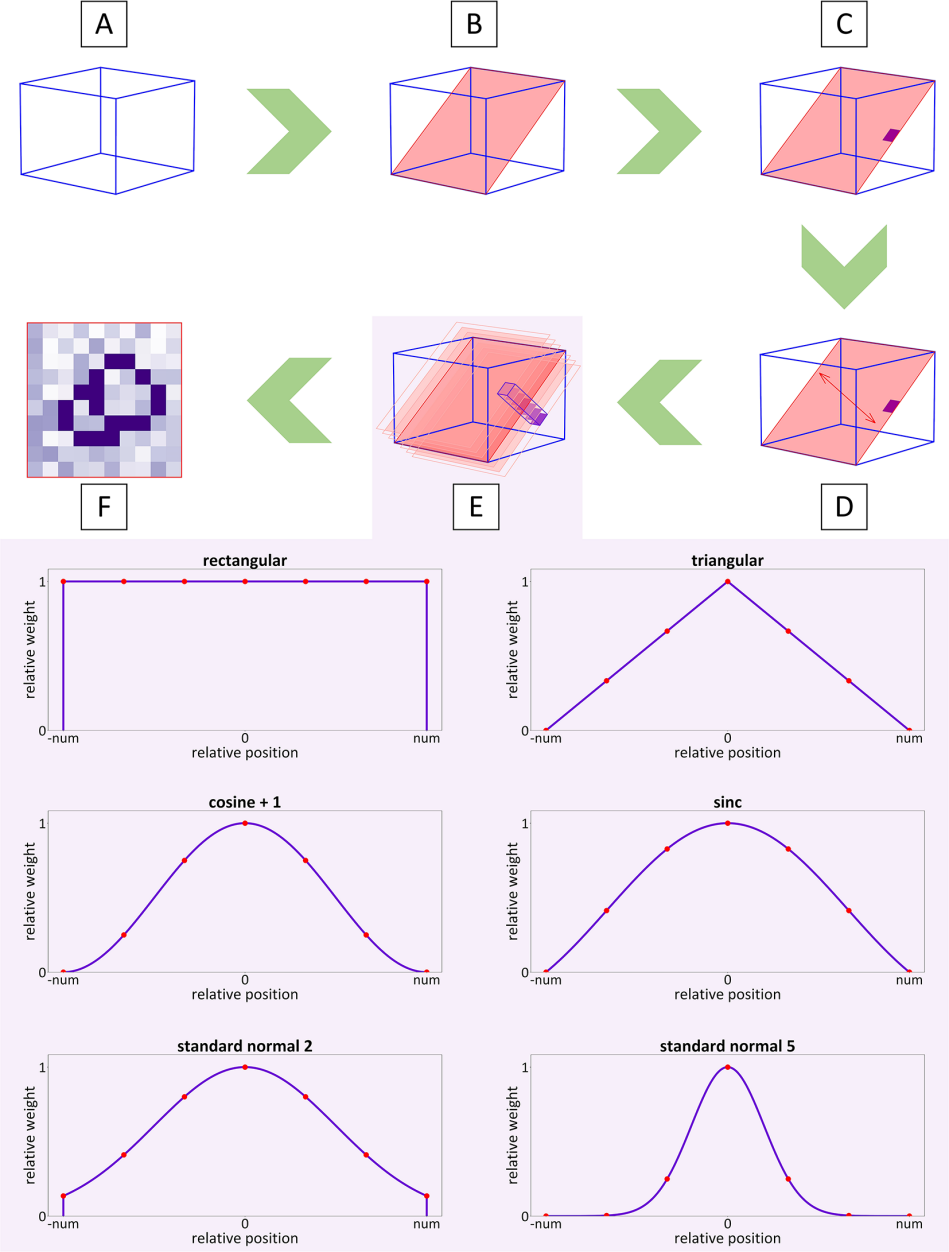
Reslice3Dto2D workflow - The acquired 3D volume (**A**) contains or is partly intersected by the reference 2D plane (**B**). The pixel location represents a discretized rectangular area (**C**). The slice thickness is incorporated by along the orthogonal normals (**D**) shifted parallel slices (**E**), such that a cubid volume arises (**E**). The totalization of the parallel located interpolated values is weighted according to the chosen slice profile (**E**) resulting in a reformatted 2D image with interpolated values from the 3D volume (**F**)

The basic principle of the Reslice3Dto2D tool is a value interpolation of 2D reference plane pixel locations in a 3D regular grid. Technically two subsequent interpolations are necessary: the in-plane space discretization and the non-unitary and potentially neither rectangular shaped through-plane slice thickness [15]. The Reslice3Dto2D tool follows this two-step interpolation approach, which is visualized in Fig. 1 and explained in the following.

The acquired 3D volume (Fig. 1A) contains partly or fully the reference 2D slice (Fig. 1B). The in-plane discretization makes a pixel representing a rectangular area (Fig. 1C). The incorporation of a slice thickness requires the calculation of the orthogonal normal towards both directions (Fig. 1D). Along these normals parallel slices are determined and the volume element (voxel) that belong to the same locations among the parallel slices represents finally the reformatted pixel (Fig. 1E). The number of parallel slices *num* along each side are calculated as

$$\eqalign{& num = round\left( {{{slice\_thickness} \over {2 \cdot {\kern 1pt} 3D\_z\_resolution}}} \right) \cr & with{\kern 1pt} _{towards {\mkern 1mu} {\mkern 1mu} acquisition{\mkern 1mu} {\mkern 1mu} direction}^{3D\_z\_resolution{\mkern 1mu} {\mkern 1mu} ={\mkern 1mu} spacing{\mkern 1mu}  {\mkern 1mu} in{\mkern 1mu} {\mkern 1mu} the{\mkern 1mu} {\mkern 1mu} 3D{\mkern 1mu} {\mkern 1mu} data{\mkern 1mu}} \cr}$$

Consequently, the distance *dist* between those slices is

$$\,dist = {{slice\_thickness} \over {2 \cdot \,num}}$$

The interpolated values at the same location across the parallel slices are finally totalized with a weight *w* that depends on the slice profile of either rectangular, triangular, cosine + 1, sinc, standard normal 2 or standard normal 5 shape (Fig. 1E):

$$\eqalign{& valu{e_{i,j}} = round\left( {{1 \over {\sum {{\kern 1pt} _{k = - num}^{num}} {w_k}}} \cdot {\kern 1pt} \sum {{\kern 1pt} _{k = - num}^{num}} {w_k} \cdot {\kern 1pt} valu{e_{i,j,k}}} \right) \cr & with{\kern 1pt} _{k{\mkern 1mu} = {\mkern 1mu} slice{\mkern 1mu}  {\mkern 1mu} number}^{i,j{\mkern 1mu} = {\mkern 1mu} position{\mkern 1mu}  {\mkern 1mu} indeces} \cr}$$

This agglomeration collapses the parallel slices into one slice at the 2D reference plane with accordingly reformatted integer values (Fig. 1F). The slice thickness can be set in the Reslice3Dto2D application to any value between 0 mm and 99.99 mm, explicitly to the 2D slice thickness or the 3D_z_resolution. The reformatted data can be exported as DICOM [12] with new assigned series number and unique identifiers.

### Validation

The interpolation was validated on an artificial dataset. This dataset consisted of a 3D cubic volume data with an isotropic resolution of 1 mm and 11 positions along all three dimensions with the iso-center at the midpoint of the volume. The value was constant along the x and y directions and linear decreased from 100 to 0 in steps of 20 from the middle towards the upper and lower z border. Additionally, three reference 2D slices were provided. One was parallel to the x-y-plane at z = 0 (parplane), one was perpendicular to that and parallel to the x-z-plane at y = 0 (perplane) and the third one trended with an angle of 45 degrees in the y-z-plane (diagplane). All planes had a slice thickness of 2 mm and 11 positions along each dimension. Parplane and perplane had an in-plane resolution of 1 mm while diagplane had a resolution of 1 mm∙√2 mm.

The reformatted values of the three reference slices were compared to expectations that has been calculated by hand for the slice thicknesses of 0 mm, 2 mm, 2.82 mm and 4.23 mm and a rectangular slice profile. The validation dataset is provided in the supplemental material S3 to enable reproduction of the validation results.

### Testing

The Reslice3Dto2D tool was applied to research data consisting of two datasets of two distinct patient groups with in total 119 patients to proof functionality on real data in the specific use-case of CMR. Dataset 1 consisted of 53 patients and was retrieved from Fenski et al. [7], who showed high congruency between a 3D compressed sensing (CS) LGE research sequence (1.25mm^3^ isotropic resolution) and a standard 2D LGE on a 1.5T Siemens Avanto^fit^ with respect to global and segmental LGE using a prior version of Reslice3Dto2D. Dataset 2 consisted of 66 patients of an ongoing prospective study including a 3D CS LGE DIXON acquisition with fat/water separation [8]. 45 patients were examined on a 1.5T Siemens Avanto^fit^ with an isotropic resolution of 1.30mm^3^ and 21 patients were examined on a 3T Siemens Skyra^fit^ with 1.25 × 1.25 × 1.30mm^3^ non-isotropic resolution. In all 119 included patients a midventricular short axis view of a 2D LGE sequence with a slice thickness of 7 mm was chosen as the reference 2D slice. A reformatting was performed for all slice profiles on slice thicknesses of 0 mm, 7 mm and 14 mm. The impact of partial volume effects is proportional to the slice thickness which in turn lowers the image sharpness [16, 17]. The sharpness of the reformatted test data was assessed with the Frequency Domain Image Blur Measure (FM) [18] and tested using the Wilcoxon and Friedman test with a significance level of *p* ≤ 0.05 among the three slice thicknesses. The FM is evaluated in the Fourier transform representation of the image in a six-step calculation approach and provides a robust metric for the quantification of image sharpness [18]. One reformatted case of each dataset was exemplary loaded into cvi42 (Version 5.13, Circle Cardiovascular Imaging Inc.), Caas MR solutions (Version 5.2, Pie Medical Imaging BV) and Medis Suite MR (Version 4.0 Medis Medical Imaging Systems BV) to check compatibility with a proprietary post-processing software.

## Results

Following the Methods section, the results are provided for implementation, validation and testing.

### Implementation

The Reslice3Dto2D tool was successfully installed and tested on Windows 10, Windows 11 and macOS 14. The provided executable files even worked without further installation. The GUI enables a usage without the necessity of programming skills. The export of reformatted data in the DICOM [12] format worked as expected.

### Validation

**Fig. 2:**
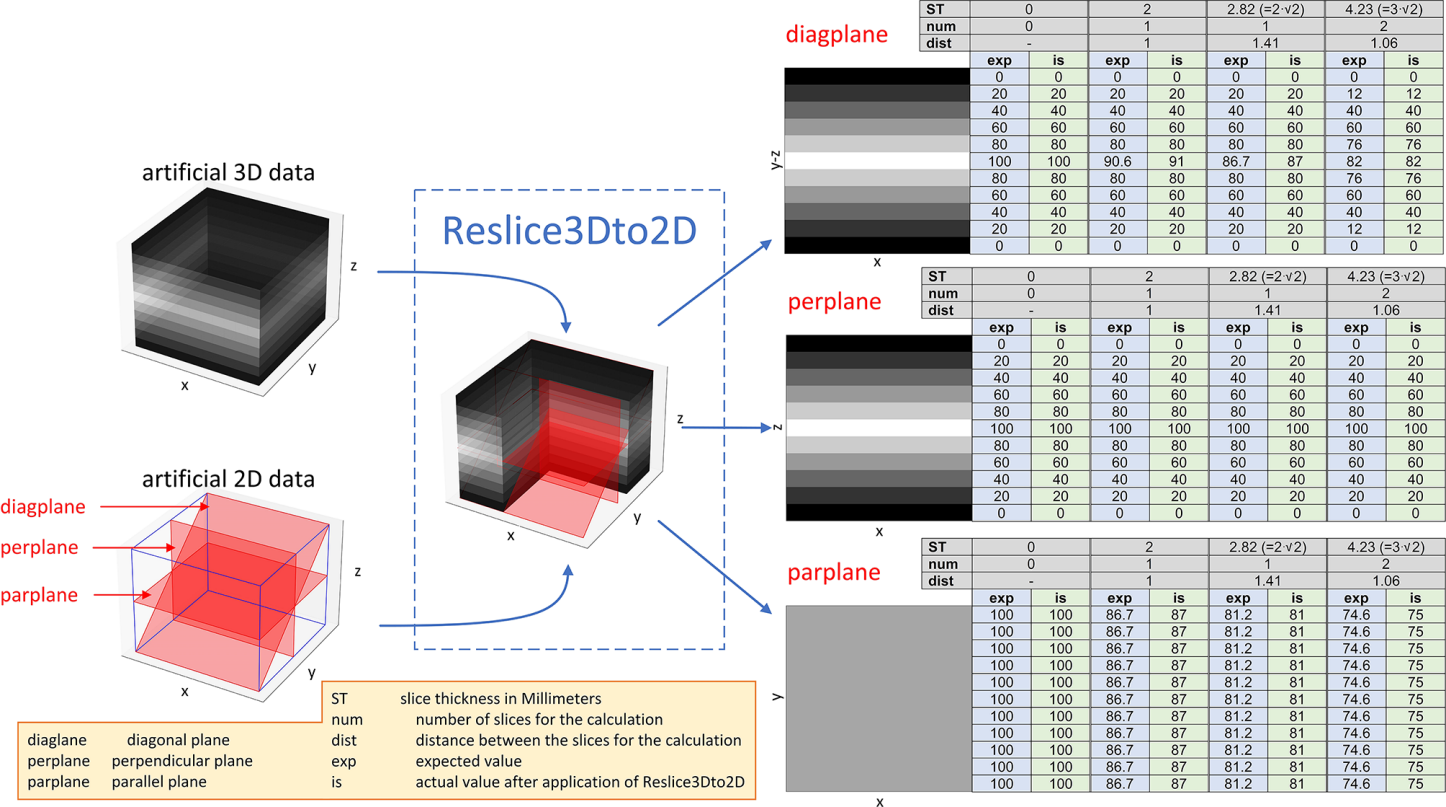
Validation data results - The artificial dataset is plotted exemplary on the left-hand side. In the middle part the actual reformatting with Reslice3Dto2D is illustrated while on the right-hand side the results are provided for the three reference 2D validation slices: diagplane, perplane and parplane for slice thicknesses of 0 mm, 2 mm, 2.82 mm and 4.23 mm and a rectangular slice profile. The actual values were compared to the expected values that had been calculated by hand

The interpolation methods used in the Reslice3Dto2D tool were verified by the artificial validation dataset. Figure 2 illustrates the reformatting of the validation data with Reslice3Dto2D and includes an overview of the numerical results that matched the expected calculations by hand.

### Testing

The test datasets were successfully reformatted and the FM significantly decreased (*p* < 0.05) for slice thicknesses of 7 mm and 14 mm compared to 0 mm. The slice profile had no impact for 0 mm slice thickness as *num* becomes zero. The slice profile standard normal 5 showed the highest and rectangular the lowest FM across the datasets for slice thicknesses greater 0 mm. This suits the expectation as partial volume effects are most present in the rectangular profile while in standard normal 5 profile the impact of parallel slices towards the borders decreases rapidly. A detailed numerical overview is shown in the table of the supplemental material S4.

**Fig. 3:**
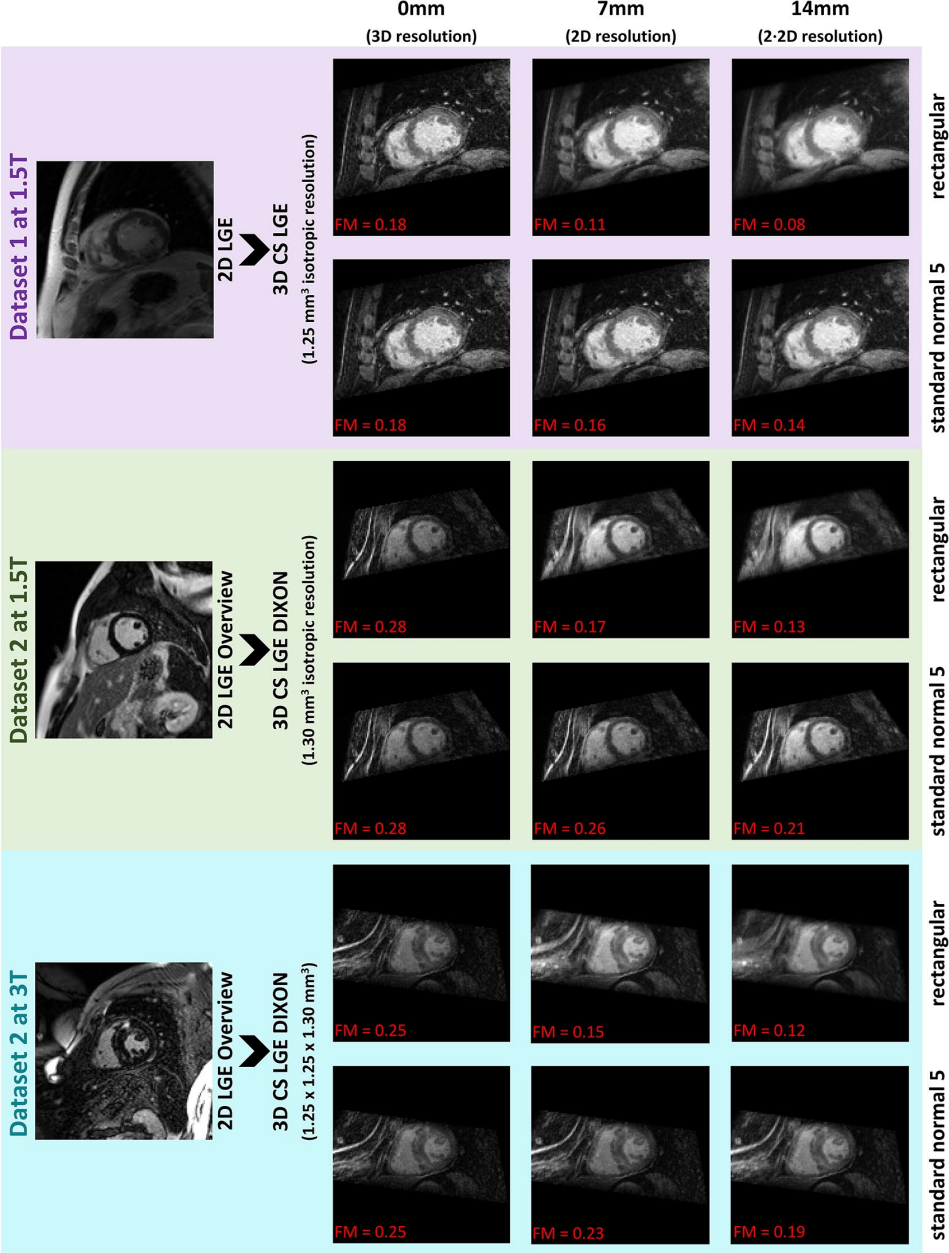
Example cases of the results of the reformatted test data - The original 2D LGE data (left side) were used as reference for the reformatting of 3D LGE data on slice thicknesses of 0 mm, 7 mm and 14 mm with rectangular and standard normal 5 slice profile (right side). For each reformatted image the Frequency Domain Image Blur Measure (FM) is provided

One case from each dataset is exemplary shown in Fig. 3 for the named extreme slice profiles: rectangular and standard normal 5 to highlight the impact of slice thickness and slice profile. All other profiles have FM values in between the shown ones. The reformatted example datasets were successfully imported into the three post-processing softwares: cvi42, CAAS MR solutions and Medis suite MR.

## Discussion

The Reslice3Dto2D tool successfully worked in all provided cases and will be discussed in the following according to the implementation, validation and testing.

### Implementation

The main use-case of the Reslice3Dto2D tool is the reformatting of 3D data at the exact same location and same pixel spacing as an acquired reference 2D image within the same examination. While MPR in commercially available software is the tool of choice to inspect the acquired 3D data for anatomical abnormalities and to hover through the structures from any perspective, the Reslice3Dto2D tool focuses on the reformatting of 3D datasets for further post-processing and quantification. Due to the simple usage in the GUI, typical long and short axis views can be extracted quickly.

While 3D slicer offers a reformat and export function as well [19], the reformatting is manually planned, which in turn is time consuming and needs some experience. Furthermore, the interpolation is solely based on the 3D data, such that slice profiles and slice thicknesses are not directly accessible. In 3D slicer [19] or with the help of VTK [20] comparable functionalities are implementable, but this requires the user to possess programming skills, whereas Reslice3Dto2D can be used by researchers in the clinical field without any programming experience after successful installation or directly with the provided executable files. A comparison of Reslice3Dto2D with manual expert reformatting on visual matching location has not been addressed in this work. This, however, may be part of a future work due to a different study design and the definition of other quality metrics.

### Validation

The functionality of the two-step interpolation in the Reslice3Dto2D tool was proven with an artificial dataset whose output suited the expected values that were calculated by hand. Although more complex artificial data is available as for example the 4D XCAT phantom [21], the manual calculation of the expected voxel values requires an inordinate high effort while the provided artificial dataset al.lowed for the requested validation in a simplified manner.

### Testing

All test datasets could be loaded and reformatted in the Reslice3Dto2D tool. As two different 3D sequences were used in the test data, the results emphasizes that the Reslice3Dto2D tool is generally applicable on 3D datasets and 2D reference slices. While 3D sequences are constantly being revised [4], it can be assumed that future 3D sequences can also be processed by the Reslice3Dto2D as long as the necessary DICOM [12] tags are provided. Although not focussed in this work and yet not been systematically studied, Reslice3Dto2D is implemented to handle also the reformatting of time-resolved 3D data like 3D CINE or 4D Flow, other 3D sequence types like 3D T1 mapping and 3D data from other imaging modalities that follow the DICOM [12] standard and include the necessary DICOM [12] tags. The software works on isotropic as well as non-isotropic 3D data. However. the acquisition of 3D data already changed the resolution from a continuous to a discretized space. Non-isotropic data is consequently more prone to sampling rate errors. Therefore, the reformatted outcome of non-isotropic 3D data needs to be treated with caution if compared to other sequences. Due to the DICOM [12] export functionality, further post-processing in conventional tools is enabled. However, the exported reformatted DICOM [12] data is based on the loaded 3D and reference 2D data. Consequently, if the original data is not importable into third party post-processing software, the reformatted data will most likely not either.

The decreasing FM in the test data with increasing slice thickness reflects the expected partial volume effect that results in a blurring of the image [16, 17]. This further validates the functionality of the tool. If the investigation of the partial volume effect is of interest, the Reslice3Dto2D software may be a useful tool to analyse this effect on available 3D data and reference 2D slices.

Other than commercial available software, Reslice3Dto2D reformats 3D data according to reference 2D data instead of a manual positioning [4, 5, 8]. This guarantees for the exact same location assuming no significant patient movement. Depending on the protocol, the scan time in CMR requires around 30 min [22]. If the 3D dataset is acquired with a high delay to the reference 2D slice, the patient may move in-between resulting in a location mismatch between the two acquisitions. In this case, classical MPR functionality is inevitable.

Although first attempts exist for 3D scar measurements in in-vitro mouse heart examinations [23], a full 3D quantitative analysis software tool is currently, to the best of our knowledge, not available for CMR. Therefore, a comparison of two distinct 3D acquisitions is enabled by reformatting both to the same 2D slice and using conventional 2D analysis solutions.

### Limitation

The used datasets contained only LGE data that were acquired on Siemens scanners with isotropic or nearly isotropic resolution, other sequence types and manufacturers were not included and, thus, limits the generalizability of the results. The Reslice3Dto2D tool is an extension of currently available software solutions for the inspection of 3D data and does not substitute MPR functionalities. A direct comparison to other tools including the definition of appropriate quality metrics is currently missing. A manual correction of the 2D plane is not possible and the in-plane pixel spacing is fixed. A reference 2D plane is a must while patient movement during the examination or different phase acquisition are not repairable.

## Conclusions

In conclusion, the Reslice3Dto2D is introduced as an easy-to-use research software tool, which is located between the examination and post-processing of 3D data if quantification is needed. It enables multiple research opportunities: perform quantification in 3D acquired sequences, comparing novel 3D data with conventional 2D acquisitions, comparing two different 3D acquisitions on the basis of equal located 2D slices and analysing the impact of the slice thickness on the quantitative outcome. The usage of the software is platform independent and can be used by researchers with different levels of experience due to the integrated GUI.

## Electronic supplementary material

Below is the link to the electronic supplementary material.

Supplementary Material 1

Supplementary Material 2

Supplementary Material 3

Supplementary Material 4

## Data Availability

Data cannot be shared publicly because of the institutional law. The data can be retrieved in an anonymized manner on reasonable request from the first (DV: darian-steven.viezzer@charite.de) or last (JSM: jeanette.schulz-menger@charite.de) author after agreement of the authorities. The source code can be accessed via the project homepage https://github.com/DSV-CUB/Reslice3Dto2D, the supplemental material S1 or zenodo(14). The packed, direct executable files for Windows and macOS are only available on zenodo(14) due to file size restrictions.

## Abbreviations

2D: Two dimensional
3D: Three dimensional
CMR: Cardiovascular magnetic resonance
CS: Compressed sensing
diagplane: Diagonal plane that bears in x- and y-z -direction
FM: Frequency Domain Image Blur Measure
GUI: Graphical user interface
Parplane: Plane that bears in x- and y--direction
LGE: Late Gadolinium Enhancement
MPR: Multi-planar reformatting
Perplane: Plane perpendicular to parplane that bears in x- and z-direction
ST: Slice thickness
