# Supplementary material for: Reslice3Dto2D: Introduction of a software tool to reformat 3D volumes into reference 2D slices in cardiovascular magnetic resonance imaging
Source: BMC Res Notes. 2024 Sep 17;17:270. doi: 10.1186/s13104-024-06931-4 (PMC11409793; doi:10.1186/s13104-024-06931-4)
Supplement: Supplementary file 1 — Supplementary Material 1 [file 13104_2024_6931_MOESM1_ESM.zip › Reslice3Dto2D_UserManual_v3.1.pdf]

Reslice3Dto2D

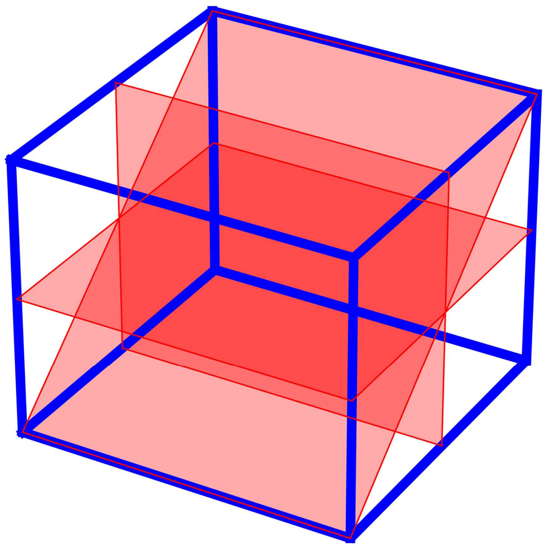

USER MANUAL

Version 3.1

Authors: Darian Viezzer, Maximilian Fenski, Thomas Hiroshi Grandy, Johanna Kuhnt, Thomas Hadler, Steffen Lange, Jeanette Schulz-Menger

Project Homepage: https://github.com/DSV-CUB/Reslice3Dto2D

Version: 3.1

Date: 08th of August 2024

License: Copyright (c) 2024 Darian Steven Viezzer,
Charité – Universitätsmedizin Berlin

Permission is hereby granted, free of charge, to any person obtaining a copy of this software and associated documentation files (the "Software"), to deal in the Software without restriction, including without limitation the rights to use, copy, modify, merge, publish, distribute, sublicense, and/or sell copies of the Software, and to permit persons to whom the Software is furnished to do so, subject to the following conditions:

The above copyright notice and this permission notice shall be included in all copies or substantial portions of the Software.

THE SOFTWARE IS PROVIDED "AS IS", WITHOUT WARRANTY OF ANY KIND, EXPRESS OR IMPLIED, INCLUDING BUT NOT LIMITED TO THE WARRANTIES OF MERCHANTABILITY, FITNESS FOR A PARTICULAR PURPOSE AND NONINFRINGEMENT. IN NO EVENT SHALL THE AUTHORS OR COPYRIGHT HOLDERS BE LIABLE FOR ANY CLAIM, DAMAGES OR OTHER LIABILITY, WHETHER IN AN ACTION OF CONTRACT, TORT OR OTHERWISE, ARISING FROM, OUT OF OR IN CONNECTION WITH THE SOFTWARE OR THE USE OR OTHER DEALINGS IN THE SOFTWARE.

Introduction

This is the User Manual for the Reslice3Dto2D software that enables the user to reformat 3D data into 2D slices at the exact same location as a reference 2D acquisition including options to specify the slice thickness and slice profile.

Graphical User Interface

The Reslice3Dto2D tool includes a graphical user interface (GUI) to facilitate the usage. In the following the GUI is shown; detailed information about functionalities behind the controls is given in the upcoming section Working Process. The main window, which appears after staring the application is shown in Figure 1.

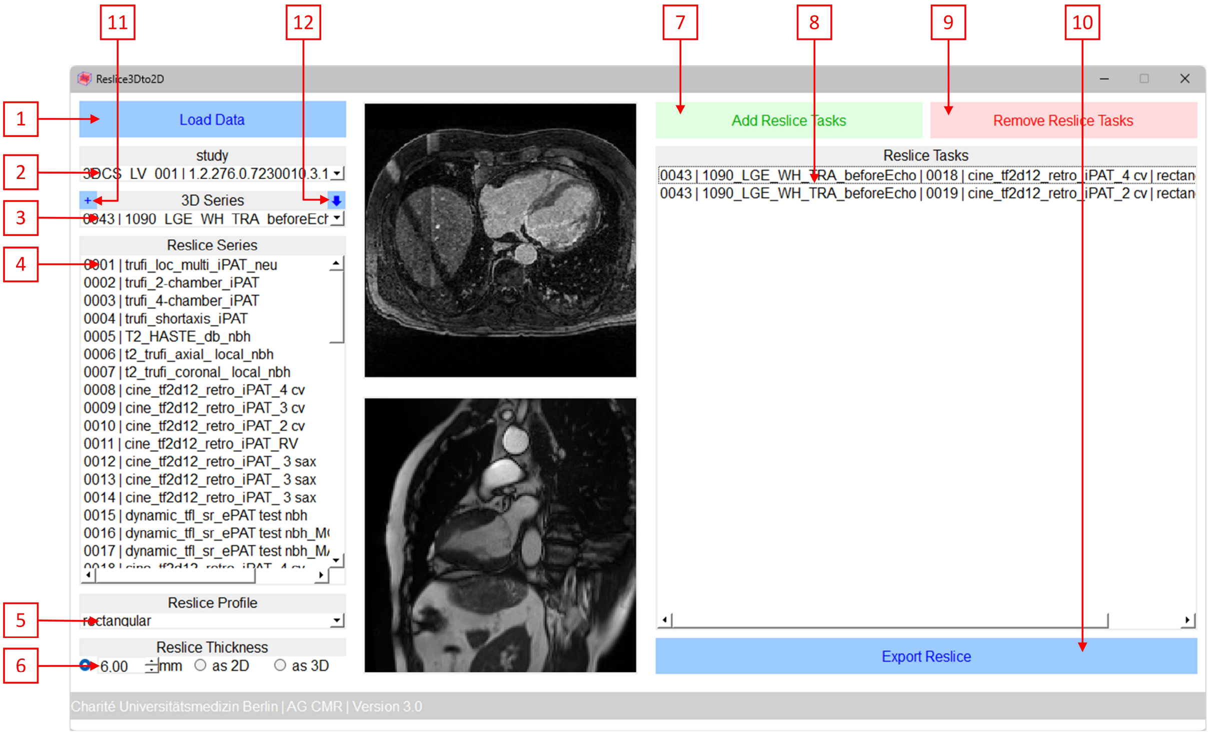

Figure 1: Reslice3Dto2D graphical user interface (GUI)

Apart from the main window, a dialog window opens, when using the button [11] as shown in Figure 2.

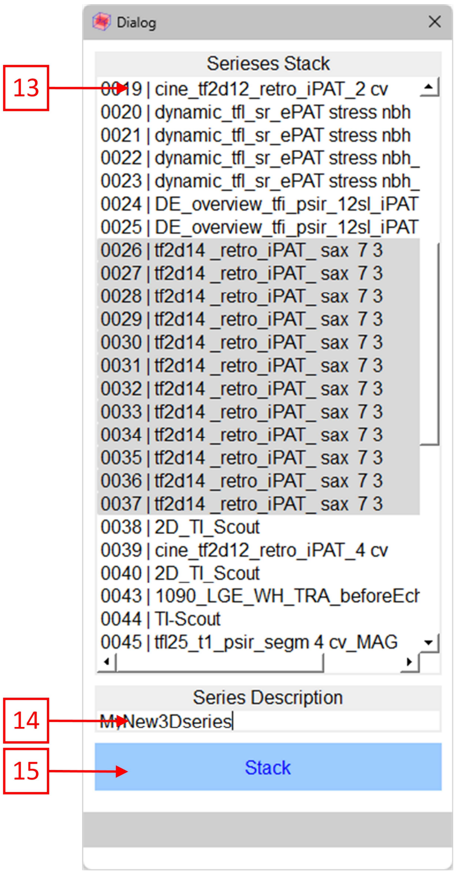

Figure 2: Reslice3Dto2D stack data to a 3D set

Working Process

In the following the usage of the Reslice3Dto2D is described in a Question-&-Answer (Q&A) style with references to the highlighted controls in the Graphical User Interface section. The Q&As are arranged in order to work like a step-by-step guide.

How to install the Reslice3Dto2D software?

The software can be directly used on Windows via the Reslice3Dto2D_Windows.exe and on macOS via the Reslice3Dto2D_macOS executable file. Alternatively, the software can be installed according to the instructions in the README.md on the GitHub homepage of the software.

How to start the Reslice3Dto2D software?

A double click on Reslice3Dto2D_Windows.exe, for Windows operating systems, or Reslice3Dto2D_macOS executable file, for macOS will start the application. If the software was installed alternatively according to the README.md, the software can be started via the RUN_Windows.bat and RUN_macOS.command respectively.

How to load data into the Reslice3Dto2D software?

The Load Data button [1] opens a select-directory-dialog. A directory with DICOM data must be selected otherwise nothings happens. The file ending is irrelevant; every file is checked for being a DICOM file. If choosing a directory with a lot of data (multiple examinations, other data than DICOM) it may take some time to load. The GUI is freezing during the loading. It is recommended to load a single or only a few examinations at once. If the loaded data includes multiple examination, then the study selection [2] appears, otherwise it is hidden. The tool automatically detects 3D data and lists them in a drop-down menu [3]. The reslice

serieses list [4] shows all serieses of an examination. A prerequisite for the DICOM data is the existence of the following DICOM tags: (0x0020, 0x000d) study UID, (0x0010, 0x0010) patient name, (0x0020, 0x0011) series number, (0x0008, 0x103e) series description, (0x0020, 0x0013) instance number, (0x0018, 0x0023) acquisition type, (0x0020, 0x0032) image position, (0x0020, 0x0037) image orientation and (0x0018, 0x0050) slice thickness.

How to switch between examinations if multiple were loaded at once?

The study selection [2] enables the user to switch between the studies and provides the patient's name as well as the study UID for orientation.

How to select a 3D sequence for the reformatting?

The 3D series drop-down menu [3] enables the user to choose a 3D sequence.

How to select a reference 2D sequence for the reformatting?

Any series can be used as the reference 2D series, also 3D or 4D Flow serieses. By selecting the according series in the list [4], the series is selected. Multiple serieses can be selected at once, if the same 3D series should be reformatted according to multiple reference orientations.

How to specify reformatted slice properties?

The slice profile [5] and the slice thickness [6] can be defined. The latter is either explicitly set in Millimeters, read from the reference slices (as 2D) or read from the selected 3D data (as 3D). As 3D data is stored in a stack of slices, the distance between those 3D slices represents the slice thickness and not the in-plane pixel spacing. This is relevant for non-isotropic 3D data. For the slice profile the following options are available: rectangular, triangular, cosine + 1, sinc, standard normal 2 and standard normal 5.

How to add a reformatting task?

After selecting the 3D series, all reference 2D slices, the according profile and slice thickness, the reformatting tasks can be added [7] to the task list [8]. This does not perform the reformatting; it only collects all intended reformatting tasks. If multiple 3D serieses exist and should be reformatted, all tasks can be added cumulative to the task list.

How to remove a reformatting task?

Individual tasks can be excluded by selecting the task in the list [8] and click on the remove button [9].

How to perform the reformatting?

If all tasks are collected, then the reformatting is performed and exported via the Export Reslice button [10]. The tool exports the reformatted data in DICOM format by using a copy of the original 2D slice and overwriting the series number, series description, protocol name, trigger time, instance number, series instance UID, SOP instance UID and media storage SOP instance UID. It is important to load the complete case into the tool, as otherwise the generated series number may collide with an already assigned one. The series description and protocol name include R3D2D at the beginning to mark that it was reformatted with Reslice3Dto2D. Each reformatting task is a new series number assigned. The exported DICOM data has a dcm file extension.

How is static 3D data reformatted with static 2D reference data?

Reformatting static 3D data by static 2D reference data is the typical use-case. This creates for each 2D slice an according single slice.

How is static 3D data reformatted with time resolved 2D reference data (like 2D CINE)?

The Reslice3Dto2D tool is aware of the slice location and phase. Time-resolved 2D data, like 2D CINE, is acquired at the same location, consequently the reformatting exports a single slice series.

How is time resolved 3D data (like 3D CINE, 4D Flow) reformatted with static 2D reference data?

The Reslice3Dto2D tool is aware of the slice location and phase. Reformatting time-resolved 3D data with static 2D reference acquisitions will create a time-resolved 2D series (like a 2D CINE, 2D Flow).

How is time resolved 3D data (like 3D CINE, 4D Flow) reformatted with time-resolved 2D reference data (like 2D CINE)?

The Reslice3Dto2D tool is aware of the slice location and phase. Reformatting time-resolved 3D data with time-resolved 2D reference acquisitions will create a time-resolved 2D series (like a 2D CINE, 2D Flow). The time resolution reflects the timing of the 3D data.

How is 3D data reformatted with 3D reference data?

The reformatting of 3D data bv another 3D data works the same as with 2D data with the only difference, that the exported series does not contain 2D but 3D data, either static or time resolved in dependence of the provided 3D data.

How to stack parallel 2D slices to a 3D stack?

An additional function of the Reslice3Dto2D tool is the stacking to a 3D dataset. For this, the + button [11] must be clicked to open the stacking dialog. In this, serieses from the list [13] can be chosen to be stacked to a 3D dataset. This forces some pre-requisites that are automatically checked: parallelism, same number of phases, same image size, equal distance among the parallel slices and upper left corner on a straight line perpendicular to the planes. If a series name is assigned [14] and the stacking is initiated [15], then a new series is created as a 3D dataset and will pop-up in the 3D series drop-down menu [3]. It is important to stack before performing the reslicing as otherwise the series number may be assigned twice. The stacked data can be used for reformatting, however, in case of parallel imaging or subsequent measurements of a short axis stack, the stacked data may not align in the phase and/or have a significant lower resolution in the third dimension than the in-plane pixel spacing.

How to export stacked 3D data?

The created 3D stack can be exported for further post-processing [12].

What is the exported file format?

All exported data is formatted as DICOM data with a .dcm file extension. The DICOM tag information are retrieved intrinsic from the loaded data.
