# Supplementary figures and images for: Reslice3Dto2D: Introduction of a software tool to reformat 3D volumes into reference 2D slices in cardiovascular magnetic resonance imaging
Source: BMC Res Notes. 2024 Sep 17;17:270. doi: 10.1186/s13104-024-06931-4 (PMC11409793; doi:10.1186/s13104-024-06931-4)

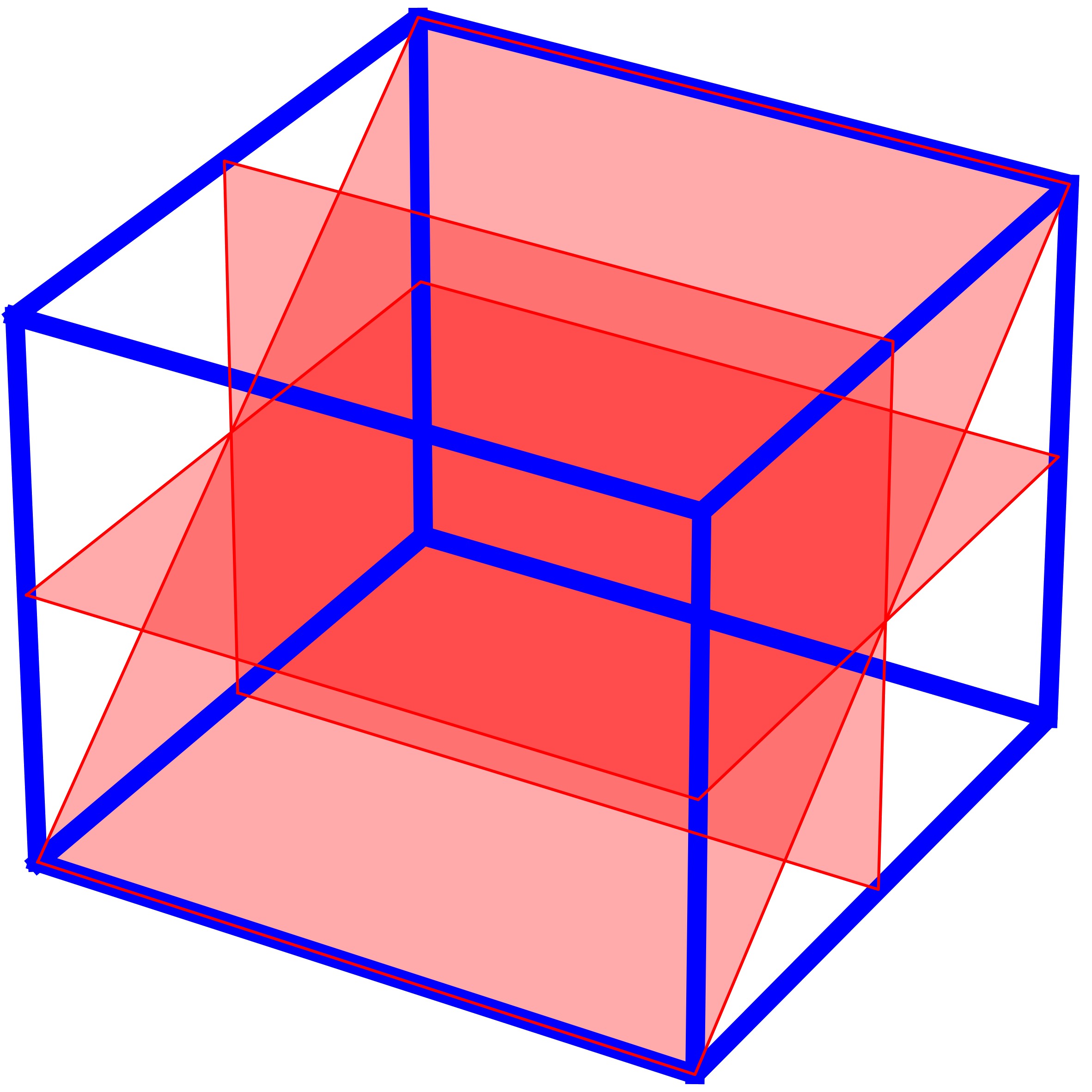

Supplement: Supplementary file 1 — Supplementary Material 1 [file 13104_2024_6931_MOESM1_ESM.zip › sourcecode/logo.png]
