# Supplementary material for: Reslice3Dto2D: Introduction of a software tool to reformat 3D volumes into reference 2D slices in cardiovascular magnetic resonance imaging
Source: BMC Res Notes. 2024 Sep 17;17:270. doi: 10.1186/s13104-024-06931-4 (PMC11409793; doi:10.1186/s13104-024-06931-4)
Supplement: Supplementary file 4 — Supplementary Material 4 [file 13104_2024_6931_MOESM4_ESM.docx]

S4 - Results of the reformatted test data

| **Dataset 1 at 1.5T: 53 patients** | | | | | | |
| --- | --- | --- | --- | --- | --- | --- |
| **SP \| ST in mm** | **0 (=3D ST)** | | **7 (=2D ST)** | | **14 (=2∙2D ST)** | |
| rectangular | 0.17±0.03 | | 0.10±0.02* | | 0.07±0.02* | |
| triangular | 0.17±0.03 | | 0.12±0.03* | | 0.09±0.02* | |
| cosine + 1 | 0.17±0.03 | | 0.12±0.03* | | 0.09±0.02* | |
| sinc | 0.17±0.03 | | 0.12±0.03* | | 0.09±0.02* | |
| standard normal 2 | 0.17±0.03 | | 0.11±0.03* | | 0.08±0.02* | |
| standard normal 5 | 0.17±0.03 | | 0.15±0.03* | | 0.12±0.03* | |
| **Dataset 2 at 1.5T: 45 patients** | | | | | | |
| **SP \| ST in mm** | **0 (=3D ST)** | | **7 (=2D ST)** | | **14 (=2∙2D ST)** | |
| rectangular | 0.24±0.05 | 0.27±0.03 | 0.15±0.04* | 0.17±0.03* | 0.12±0.04* | 0.13±0.03* |
| triangular | 0.24±0.05 | 0.27±0.03 | 0.19±0.05* | 0.21±0.03* | 0.14±0.04* | 0.15±0.03* |
| cosine + 1 | 0.24±0.05 | 0.27±0.03 | 0.19±0.05* | 0.21±0.03* | 0.15±0.04* | 0.16±0.03* |
| sinc | 0.24±0.05 | 0.27±0.03 | 0.18±0.05* | 0.20±0.03* | 0.14±0.04* | 0.15±0.03* |
| standard normal 2 | 0.24±0.05 | 0.27±0.03 | 0.18±0.05* | 0.19±0.03* | 0.13±0.04* | 0.15±0.03* |
| standard normal 5 | 0.24±0.05 | 0.27±0.03 | 0.22±0.05* | 0.24±0.03* | 0.19±0.05* | 0.21±0.03* |
| **Dataset 2 at 3T: 21 patients** | | | | | | |
| **SP \| ST in mm** | **0 (=3D ST)** | | **7 (=2D ST)** | | **14 (=2∙2D ST)** | |
| rectangular | 0.23±0.04 | 0.26±0.03 | 0.14±0.03* | 0.17±0.02* | 0.11±0.02* | 0.13±0.02* |
| triangular | 0.23±0.04 | 0.26±0.03 | 0.17±0.03* | 0.20±0.02* | 0.13±0.03* | 0.15±0.02* |
| cosine + 1 | 0.23±0.04 | 0.26±0.03 | 0.18±0.03* | 0.21±0.03* | 0.14±0.03* | 0.16±0.02* |
| sinc | 0.23±0.04 | 0.26±0.03 | 0.17±0.03* | 0.20±0.02* | 0.13±0.03* | 0.15±0.02* |
| standard normal 2 | 0.23±0.04 | 0.26±0.03 | 0.16±0.03* | 0.19±0.02* | 0.12±0.03* | 0.15±0.02* |
| standard normal 5 | 0.23±0.04 | 0.26±0.03 | 0.20±0.04* | 0.23±0.03* | 0.17±0.03* | 0.20±0.02* |

*The mean ± std of the Frequency Domain Image Blur Measure (FM) for different slice thicknesses (ST) is provided for each implemented slice profile (SP) for each test dataset. In test dataset 2 grey values represent reformatted fat and blue values represent reformatted water images; statistically significance is denoted with an * (p<0.05) with respect to a slice thickness of 0mm.*
